# Exosomes of Epstein-Barr Virus-Associated Gastric Carcinoma Suppress Dendritic Cell Maturation

**DOI:** 10.3390/microorganisms8111776

**Published:** 2020-11-12

**Authors:** Munetoshi Hinata, Akiko Kunita, Hiroyuki Abe, Yasuyuki Morishita, Kei Sakuma, Hiroharu Yamashita, Yasuyuki Seto, Tetsuo Ushiku, Masashi Fukayama

**Affiliations:** 1 Department of Pathology, Graduate School of Medicine, The University of Tokyo, Tokyo 113-0033, Japan; fircothep262@gmail.com (M.H.); kunita@m.u-tokyo.ac.jp (A.K.); hiroyuki.abe.1101@gmail.com (H.A.); moriyasu@m.u-tokyo.ac.jp (Y.M.); k-sakuma@m.u-tokyo.ac.jp (K.S.); usikut@gmail.com (T.U.); 2 Department of Gastrointestinal Surgery, Graduate School of Medicine, The University of Tokyo, Tokyo 113-0033, Japan; hiroharu.yamashita@gmail.com (H.Y.); seto-tky@umin.ac.jp (Y.S.); 3 Asahi TelePathology Center, Asahi General Hospital, Asahi City, Chiba 289-2511, Japan

**Keywords:** Epstein-Barr virus, gastric cancer, exosome, dendritic cells

## Abstract

The Epstein-Barr virus (EBV)-associated gastric carcinoma (EBVaGC) is characterized by the infiltration of lymphocytes and a unique tumor microenvironment. Exosomes from cancer cells are essential for intercellular communication. The aims of this study were to investigate the secretion of EBVaGC exosomes and their physiological effect on dendritic cell maturation in vitro and to characterize dendritic cells (DCs) in EBVaGC in vivo. Western blotting analysis of CD63 and CD81 of exosomes from EBV-infected gastric cancer cell lines indicated an increase in exosome secretion. The fraction of monocyte-derived DCs positive for the maturation marker CD86 was significantly suppressed when incubated with exosomes from EBV-infected gastric cancer cell lines. Immunohistochemical analysis of GC tissues expressing DC markers (S100, Langerin, CD1a, CD83, CD86, and BDCA-2) indicated that the density of DCs was generally higher in EBVaGC than in EBV-negative GC, although the numbers of CD83- and CD86-positive DCs were decreased in the group with high numbers of CD1a-positive DCs. A low number of CD83-positive DCs was marginally correlated with worse prognosis of EBVaGC in patients. EBVaGC is a tumor with abundant DCs, including immature and mature DCs. Moreover, the maturation of DCs is suppressed by exosomes from EBV-infected epithelial cells.

## 1. Introduction

The Epstein-Barr virus (EBV) is a γ-herpesvirus and has been associated with neoplastic growth of various human cells, including lymphocytes of B-, T-, and NK-cell lineages and epithelial cells of the nasopharynx and stomach [1]. How EBV initiates and maintains neoplastic growth of latently infected cells that counteract immune surveillance in a specific milieu of each cell type has not yet been elucidated. EBV-associated gastric carcinoma (EBVaGC) is characterized by the clonal growth of EBV-infected stomach epithelial cells, and the frequency of EBVaGC takes up nearly 10% of gastric carcinoma cases [2]. Many cases of gastric carcinoma are treated by endoscopy and surgery; however, 5-year survival rates still range from 30% to 70% [3]. EBVaGC has a unique histologic subtype that is known as gastric carcinoma with lymphoid stroma (GCLS); EBVaGC requires intensive investigation such that its interaction with immune cells, especially in this type of EBV-infected malignant tumor, is elucidated.

The tumor microenvironment, which consists of inflammatory cells, stromal cells, vasculatures, and extracellular matrix, is an indispensable factor in the pathophysiology of cancers. Inflammatory cells include lymphocytes macrophages, neutrophils, and dendritic cells (DCs) [4]. During the development of the tumor microenvironment, intercellular communications are accomplished through the secretion of proteins and nucleotides and the various forms of cell-to-cell contact such as ligand-receptor interactions on the cell surface. Recently, exosomes or extracellular vesicles from cancer cells were reported as important tools for communication [5]. The elucidation of the characteristics of exosomes and their role in tumor development is expected to contribute on how the tumor microenvironment and cancer growth can be manipulated and suppressed.

Pegtel et al. [6] demonstrated that EBV-encoded microRNAs (miRNAs) are secreted by EBV-infected B cells through exosomes. EBV-miRNAs are internalized by non-infected dendritic cells, thereby downregulating immunoregulatory genes. Dendritic cells, which conduct acquired immunity, are also abundant in EBVaGC; however, their significance has not yet been fully elucidated [7,8,9,10]. The aims of this study were to (1) characterize the physical properties of EBVaGC exosomes, (2) identify their physiological effects on dendritic cell maturation in vitro, and (3) evaluate dendritic cells in EBVaGC in vivo. In the present study, we employed the use of dendritic cell markers suitable for immunohistochemical analysis as previously reported [11] to visualize tumor-associated dendritic cells (TA-DCs) and bridge findings from the flow cytometry analysis of gastric cancer-derived exosomes with those of the immunohistochemical analysis of gastric cancer tissues; the dendritic cell markers we used include CD1a, which is a marker for immature monocyte-derived DCs (moDCs), CD83 and CD86 for mature or activated DCs [12], and other markers (S100, Langerin, and BDCA-2). The maturation of moDCs was considerably suppressed in EBVaGC, which might have been mediated through the excretion of exosomes from the neoplastic cells infected with EBV.

## 2. Materials and Methods

### 2.1. Culture of Gastric Cancer Cell Lines

For in vitro experiments, gastric cancer cell lines MKN7, MKN74 (Riken BioResource Center Cell Bank, Tsukuba, Japan), and SNU719 (Korean Cell Line Bank, Seoul, Korea) were used. MKN7 and MKN74 were originally derived from well and moderately differentiated gastric adenocarcinoma without EBV infection, respectively. SNU719 was derived from EBVaGC. MKN7 and MKN74 were artificially infected with neomycin-resistant recombinant EBV in our laboratory, according to a cell-to-cell contact method [13] to yield stable infected gastric cancer cell lines MKN7 + EBV and MKN74 + EBV, respectively. These cell lines were cultured in RPMI 1640 medium (Nacalai Tesque, Kyoto, Japan) supplemented with 10% fetal bovine serum (CELLect; MP Biomedicals, Irvine, CA, USA) and penicillin (100 U/mL)-streptomycin (100 µg/mL) mixed solution and incubated at 37 °C in a humidified atmosphere containing 5% CO_2_. For the culture of MKN7 + EBV and MKN74 + EBV, 50 µg/mL G-418 Solution (Roche Diagnostics, Basel, Switzerland) was also added to the culture medium.

The Burkitt lymphoma cell line Akata, used in the cell-to-cell contact method, was a gift from Prof. Kenzo Takada (HokkaidoUnivesity). Namalwa (CRL-1432) and Raji (CCL-86) were also Burkitt lymphoma cell lines, and used as a control for evaluation of EBV-DNA and latent and lytic genes of EBV. Both cell lines were purchased from American Type Culture Collection (Manassas, VA, USA). These cells were similarly cultured as described above.

### 2.2. RNA Isolation and RT-PCR

Total RNA was extracted from gastric cancer cells using ISOGENII (Nippon Gene, Tokyo, Japan). Total RNA (0.5 μg) was used for cDNA synthesis with ReverTra Ace qPCR RT Kit (Toyobo, Osaka, Japan). The mRNA levels were measured by Thermal Cycler Dice Real Time System (TaKaRa, Shiga, Japan) and KAPA SYBR Fast qPCR Kit (Kapa Biosystems, Wilmington, MA, USA). The primer sequences were as follows; *EBNA1*-Fwd: 5′-CCTCCCTGGTTTCCACCTAT -3’, *EBNA1*-Rev: 5′-TCCTCACCCTCATCTCCATC-3′, *BZLF1*-Fwd: 5′-CTGGTGTCCGGGGGATAAT-3’, *BZLF1*-Rev: 5′-TCCGCAGGTGGCTGCT-3′, *GAPDH*-Fwd: 5′-CAACGGATTTGGTCGTATTGG-3′, and *GAPDH*-Rev: 5′-GCAACAATATCCACTTTACCAGAGTTAA-3′.

### 2.3. Exosome Isolation and Purification

Exosomes were collected according to the method described by Yoshioka et al. [14] In brief, gastric cancer cell lines were washed with phosphate-buffered saline (PBS) twice and cultured in growth media (Advanced RPMI1640, Thermo Fisher Scientific, Waltham, MA, USA) without serum for 48 h, followed by the collection of the culture supernatant. There was no additional procedure to induce exosome production. Most cells were viable after the 48 h culture, and the total cell number was about 1 × 10^8^ cells for each cell line. Afterwards, 200 mL supernatant solutions from each cell line culture was centrifuged at 300× *g* for 10 min, and the resulting supernatant was subsequently centrifuged at 9240× *g* for 40 min. Then, the supernatant was filtered through a 0.22-µm cellulose acetate membrane filter (Corning, New York, NY, USA), and cell debris was thoroughly removed. The filtrate was concentrated by ultrafiltration using Amicon Ultra 15 Ultracel 50 kDa (Merck Millipore, Burlington, MA, USA), and 10–30 mL of samples was obtained. These samples were ultracentrifuged at 110,000× *g* for 70 min, and the supernatant was discarded. Then, the samples were redissolved in PBS and ultracentrifuged at 110,000× *g* for 70 min again.

### 2.4. Preparation of Whole Cell Lysates

Cultured cell lines (about 1 × 10^7^ cells for each cell line) were washed twice with PBS, and 500 μL lysis buffer was added to each culture plate that was placed on ice. Thereafter, the cells were scraped with a cell scraper and mixed by pipetting and vortexing. The mixture was allowed to stand for 30 min and centrifuged at 15,000 rpm for 30 min; the resulting supernatant was collected. For the preparation of lysates under non-reducing conditions, a lysis buffer comprising 50 mM Tris-HCl pH 7.5, 150 mM NaCl, and 1.0% NP-40 was used. For the preparation of lysates under reducing conditions, RIPA buffer comprising 50 mM Tris-HCl pH 7.5, 150 mM NaCl, 1% Triton X-100, 0.1% sodium dodecyl sulfate (SDS), 10% glycerol, 2 mM EDTA, and 10 mM NaF was used.

### 2.5. Western Blotting

Western blotting of exosomes was performed according to the recommendation (MISEV2018) [15]. Exosome samples (equivalent to the amount of exosome in 5 × 10^5^ cells per each sample) or whole cell lysates (20 μg of protein per each sample) were incubated with 6X sample buffer (350 mM Tris-HCl pH 6.8, 10% SDS, 30% glycerol, at 37 °C for 30 min for preparation under non-reducing conditions; 350 mM Tris-HCl pH 6.8, 10% SDS, 500 mM dithiothreitol [DTT], 30% glycerol, at 95 °C for 5 min for preparation under reducing conditions). The samples were transferred onto a polyvinylidene fluoride (PVDF) membrane and blocked with tris-buffered saline (TBS) with Tween 20 containing 5% skim milk. The samples were incubated with primary antibodies at 25 ± 2 °C for 2 h. Then, the samples were washed and incubated with HRP-conjugated secondary antibody at 25 ± 2 °C for 1 h. The samples were washed, and chemiluminescence signals were detected using a chemiluminescence kit (ImmunoStar Reagent or ImmunoStar LD, FUJIFILM Wako Pure Chemical Corporation, Osaka, Japan). Anti-CD63 (BD Pharmingen, San Diego, CA, USA, clone H5C6, 1:200), anti-CD81 (Santa Cruz Biotechnology, Santa Cruz, CA, USA, clone 5A6, 1:200), and anti-β-actin (A5441; Sigma-Aldrich, St. Louis, MO, USA, 1:10,000) antibodies were used as primary antibodies. Procedures were performed under non-reducing conditions for CD63 and CD81, and under reducing conditions for β-actin.

### 2.6. Transmission Electron Microscopy

A formvar membrane was placed on HF36 400 mesh (Pyser-SGI, Edenbridge, Physer-SGI, UK), dried, and carbon-deposited. The exosome sample was placed thereon, excess liquid was removed with filter paper after 2–3 s. Then, a small drop of 1% uranium acetate was applied for a few seconds; the membrane was blotted with filter paper and air-dried. The samples were observed with JEM-1400 Transmission Electron Microscope (JEOL, Tokyo, Japan).

### 2.7. Culture and Stimulation of Dendritic Cells Derived from Human Monocytes

Peripheral blood was collected from healthy donors. Blood fractions containing mononuclear cells were separated using Ficoll Paque Plus (Cytiva, Marlborough, MA, USA) according to the manufacturer’s instructions. After the blood was centrifuged at 300× *g* for 10 min at 25 ± 2 °C, the sediment was suspended in 80 μL autoMACS running buffer (Miltenyi Biotec, Bergisch Gladbach, Germany) per 1 × 10^7^ cells. Then, 20 μL CD14 MicroBeads (Miltenyi Biotec) per 1 × 10^7^ cells was added and incubated at 4 °C for 15 min. Afterwards, 1–2 mL buffer was added per 1 × 10^7^ cells, and the mixture was centrifuged at 4 °C, 300× *g* for 10 min, and the supernatant was discarded. The sediment was suspended in 500 μL buffer. Thereafter, CD14-positive monocytes were collected using a magnetic cell separator according to the manufacturer’s instructions. An aliquot was used for fluorescence-activated cell sorting (FACS), and the rest of the sample was transferred on a 6-well plate at a density of 1.5 × 10^6^ cells/5 mL/well and cultured at 37 °C in a humidified atmosphere containing 5% CO_2_; the culture medium used comprised RPMI 1640, 10% FBS, 1% penicillin-streptomycin mixed solution, IL-4 (Wako, 50 ng/mL), and GM-CSF (Wako, 50 ng/mL). Monocytes incubated in a medium without IL-4 and GM-CSF, which are used for dendritic cell differentiation, were used as control.

Exosome samples derived from the gastric cancer cell line were diluted to 20 μg/mL (protein concentration measured by using bicinchoninic acid (BCA) assay) in PBS and sterilized by filtration with 0.22 µm sterile filter (MILLEX-GV, Merck Millipore, Cork, Ireland). Subsequently, 2 μg exosomes were added to the culture on days 0, 3, and 5; on the other hand, 100 μL PBS, instead of exosomes, was added to separate cultures as controls that simulate immature DC. Furthermore, onto the cultures as a control that simulates mature dendritic cells, incubation, addition of 100 μL PBS on days 0, 3, and 5, and addition of 1 μg/mL lipopolysaccharides (Sigma-Aldrich) on day 5 were performed.

Six days after the start of the culture, the cells were detached by pipetting and scraping and were collected. After centrifugation at 1500 RPM for 10 min at 25 ± 2 °C, the supernatant was discarded, and the sediment resuspended in 205 µL PBS. The number of cells was counted and aliquoted into 50 µL solutions, to which 2.5 μL primary antibody was added; the solutions were incubated on ice for 15 min. Then, 500 μL PBS was added, and the mixture was centrifuged at 2000 RPM at 4 °C for 10 min. The supernatant was removed, and the sediment resuspended in 500 mL of PBS. This was used for analysis by FACS. The primary antibodies used were CD1a-PE (BioLegend, San Diego, CA, USA, clone HI149) and CD86-PE (TONBO Biosciences, San Diego, CA, USA, clone IT2.2).

### 2.8. Tissue Samples

Pathological records were reviewed, and formalin-fixed paraffin-embedded gastric cancer tissues were obtained from the archives of the Department of Pathology, The University of Tokyo Hospital. EBVaGC cases were collected, and EBV-negative gastric cancer cases were also included as control samples. EBV infection in cancer cells was confirmed by EBER-in situ hybridization. For immunohistochemical staining, tissue microarray data constructed from these cases were used. The study was conducted in accordance with the Declaration of Helsinki, and the protocol was approved by the Ethics Committee of the Graduate School of Medicine, the University of Tokyo (10461-(10)).

### 2.9. Immunohistochemistry

Immunohistochemical staining for Langerin, CD1a, S100, CD83, CD86, and BDCA-2 was performed on tissue microarray using VENTANA BenchMark, an automated immunostainer (Roche Diagnostics). The staining conditions were as follows: CC1 buffer for 60 min, primary antibody reaction for 32 min, and visualization with iVIEW DAB v3. The primary antibodies used were mouse monoclonal anti-Langerin antibody (Leica Biosystems, Wetzlar, Germany, clone 12D6, 1:200), mouse monoclonal anti-CD1a antibody (Agilent Technologies, Santa Clara, CA, USA, clone O10, 1:100), rabbit polyclonal anti-S100 antibody (Roche Diagnostics), mouse monoclonal anti-CD83 antibody (Santa Cruz Biotechnology, clone Hb15a, 1:100), rabbit monoclonal anti-CD86 antibody (Cell Signaling Technology, Danvers, MA, USA, clone E2G8P, 1:50), and mouse monoclonal anti-BDCA-2 antibody (DENDRITICS, Lyon, France, clone 124B3.13, 1:50).

To evaluate the number of immune cells, two tissue microarray cores were used in each case of gastric carcinoma. Each core comprised approximately 4 mm^2^ tissue; therefore, a total of 8 mm^2^ tissue area was evaluated for each case. Tissue slides were digitized using a Nanozoomer 2.0-HT virtual slide scanner (Hamamatsu Photonics, Hamamatsu, Japan), and tumor areas were measured using NDP.view2 software (Hamamatsu Photonics). The area containing viable tumor cells was considered the tumor area. All tumor areas were specified and measured for each tissue microarray core. The ratio of tumor area to whole tissue area differed from case to case (from 5% to 100%). The dendritic cells expressing each marker (Langerin, CD1a, S100, CD83, CD86, and BDCA-2) that infiltrated the tumor areas were visually counted under an optical microscope (ECLIPSE 80i, Nikon, Tokyo, Japan). As some dendritic cells have a morphologically complex cytoplasm, a cell whose nucleus was recognizable was counted as a distinct cell. Among S100-positive cells, those considered to be morphologically macrophages were excluded. The density of DCs was calculated by dividing the cell number by the area of the tumor.

### 2.10. Analysis of Correlation between the Density of Dendritic Cells and Clinicopathological Characteristics

Gastric cancer cases were divided into subgroups according to clinicopathological parameters, including age, sex, tumor size, Lauren classification, tumor depth, venous invasion, lymphatic invasion, and lymph node metastasis. The mean value of the density of DCs was calculated for each subgroup. Gastric cancer cases were also divided into two groups according to the density of DCs that expressed each marker, and overall survival was compared using the Kaplan–Meier method. As a threshold of DC density, the median value in each subgroup of EBVaGC and EBV-negative gastric cancer, respectively, was used, as DC density differed greatly between EBVaGC and EBV-negative gastric cancer.

### 2.11. Statistical Analysis

For the comparison of DC density that infiltrated gastric cancer tissue and the comparison of exosome size on transmission electron microscopy, Wilcoxon rank sum test was used. For the comparison of overall survival, log-rank test was used. Statistical analysis was performed using R software (version 3.2.3; R Foundation for Statistical Computing, Vienna, Austria) [16].

## 3. Results

### 3.1. Exosomes Purified from Gastric Cancer Cell Lines with and without EBV Infection

**Figure 1:**
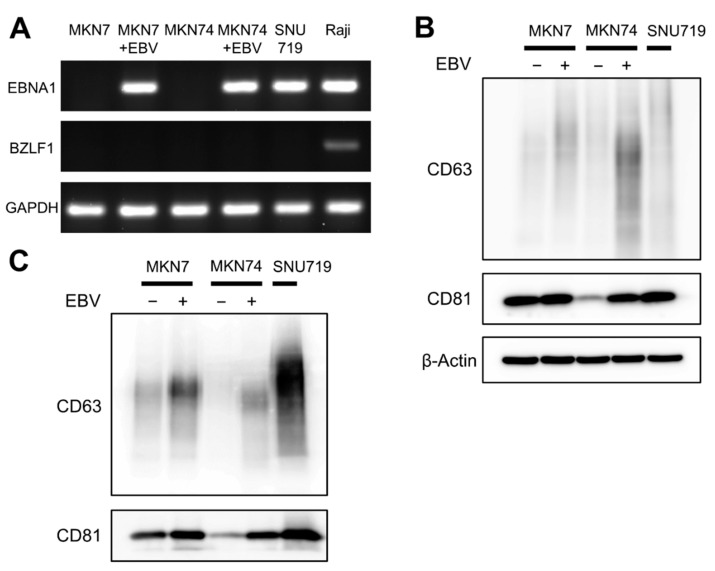
Detection of exosome marker proteins in gastric cancer cell lines with and without EBV-infection (MKN7, MKN7 + EBV, MKN74, and MKN74 + EBV) and the gastric cancer cell line of EBVaGC (SNU719). (**A**): PCR evaluation of an EBV-latent gene, *EBNA1* and RT-PCR evaluation of a lytic gene, *BZLF1*. (**B**,**C**): Western blot analysis of exosome marker proteins in cell lysates (**B**) and exosomes (**C**). Proteins from the same amount of cell lysate (20 μg of protein per each sample) or exosomal preparations from the same number of cells from gastric cancer cell lines (equivalent to the amount of exosome in 5 × 10^5^ cells per each sample) were separated by SDS-PAGE, transferred onto a PVDF membrane, and incubated with specific antibodies (CD63, CD81, β-Actin). Signals were detected with HRP-conjugated secondary antibody and by using a chemiluminescence kit.

To evaluate the exosomes of gastric cancer cell lines with EBV-infection (MKN7 + EBV and MKN7 + EBV) and of a cancer cell lines from an EBV gastric carcinoma (SNU719), the presence of EBV was confirmed by positive results of *EBNA1*-RT-PCR and the absence of lytic infection by negative results of *BZLF1*-RT-PCR, respectively (Figure 1A). Then, exosomes were extracted from the culture media of MKN7, MKN7 + EBV, MKN74, MKN7 + EBV, and SNU719 by ultracentrifugation.

CD63 and CD81, which are exosome marker proteins, were evaluated in cell lysates and exosome samples from gastric cancer cell lines (Figure 1). Increased levels of CD63 and CD81 were observed in both cell lysates and exosome samples in MKN7 + EBV and MKN74 + EBV, respectively, compared to those in MKN7 and MKN74. The amount of CD63 and CD81 were much greater in SNU719 than in MKN7 + EBV and MKN74 + EBV.

**Figure 2:**
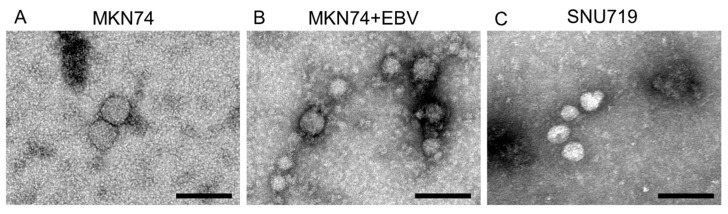
Characterization of exosomes by transmission electron microscopy (**A**–**C**). Exosomes were extracted from culture supernatant of gastric cancer cell lines by ultracentrifugation. Exosomes were recognized as small vesicles 30–100 nm in diameter under transmission electron microscopy. (**A**) MKN74, (**B**) MKN74 + EBV, (**C**) SNU719. Scale bar: 100 nm.

The integrity of purified exosomes was confirmed by transmission electron microscopy as small vesicles with 30–100 µm diameter (Figure 2A–C) were observed. The diameters of exosomes from MKN74, MKN74 + EBV, and SNU719 were 71.3 ± 43.7 nm (n = 6), 33.9 ± 8.5 nm (n = 25), and 69.6 ± 42.9 nm (n = 29), respectively. The diameter of the exosomes in MKN74 + EBV was significantly smaller than that in MKN74 (*p* < 0.0001, determined by using Wilcoxon rank sum test).

### 3.2. Effect of Artificial EBV-Infection on Exosomes of Gastric Cancer Cells

To evaluate the effect of artificial EBV infection, western blotting and transmission electron microscopy were performed (Table 1). The effects of EBV infection on exosomes were almost similar with those on cell lysates. We assume a simple model as follows: (1) exosomes are spheres, (2) protein amounts are proportional to the particle volume, and (3) the densities of CD63 and CD81 on the particle surface are constant. Thus, the densities of CD63 and CD81 are proportional to the surface area of the particle surface. Since the diameter of MKN74 + EBV exosomes was nearly half of MKN74 exosomes, the number of particles was calculated as 8-fold and the protein content of each particle 0.125-fold (Appendix A).

### 3.3. Exosomes of EBVaGC Gastric Cancer Cells

The results of the evaluation of exosomes in SNU719 by Western blotting and transmission microscopy were compared to those of EBV-non-infected gastric cancer cell lines MKN7 and MKN74 (Table 2). The densities of CD63 and CD81 were nearly 2-fold higher than those of cell lysates. The fold increase in protein amounts were 1.5-fold or comparable to those of CD63 and CD81 densities, respectively. The diameters of the particles of SNU719 and MKN74 were similar, suggesting that the change in protein content in each particle was negligible and that the increase in exosome numbers was 4- to 6-fold higher in SNU719 (Appendix A).

### 3.4. FACS Analysis of Human Monocyte-Derived Dendritic Cells Incubated with Gastric Cancer Exosomes

**Figure 3:**
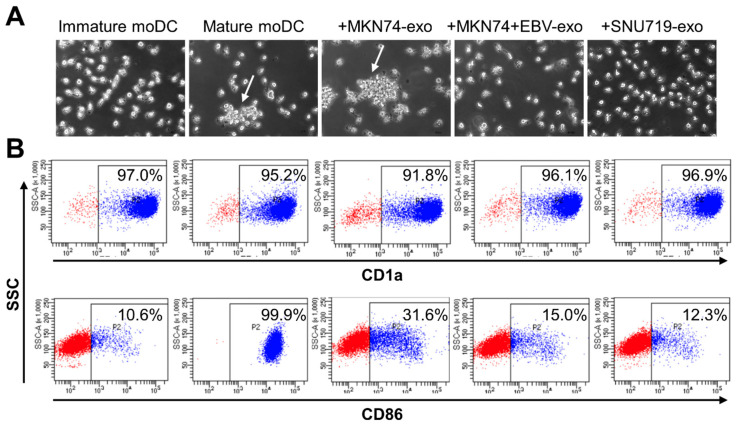
Differential effects of exosomes secreted by EBV-infected gastric cancer cells on the maturation phenotype of human monocyte-derived dendritic cells (moDCs). Immature DCs were either untreated or treated with the indicated exosomes or with LPS as a positive control for maturation. (**A**) Phase-contrast images of moDCs cultured with exosomes in EBV-infected cells. Large aggregates of cells (arrows) as reported for mature moDCs were found in MKN74-derived exome-treated moDCs but not in EBV-positive cell-derived exosome-treated cells. (**B**) Flow cytometry results of CD1a and CD86 on moDCs incubated with the indicated exosomes. The data are presented as % positive cells of indicated markers. The results are representative of three independent experiments from different donors.

Human monocyte-derived dendritic cells were cultured with exogenous exosomes of MKN74, MKN74 + EBV, and SNU719 (Figure 3A). Mature moDCs frequently formed cell aggregates, but such aggregates disappeared with the exosomes in MKN74 + EBV and SNU719.

Immature and mature markers were analyzed by FACS. When monocytes were stimulated with LPS, CD86-positive cells reached 99.9% (data not shown), thereby confirming optimal experiment conditions. In a representative experiment, moDCs were shown to be CD1a-positive. The ratios of CD86 positive cells were 15.0% and 12.3% when incubated with the gastric cancer exosomes in MKN74 + EBV and SNU719, respectively. The ratios were significantly lower than those of MKN74 exosomes (31.6%).

To exclude the possibility that a trace amount of EBV virus infected moDCs, we checked *EBNA1*-DNA in cultured monocytes, which were incubated with exosomes of SNU719. Using TaqMan quantitative PCR (qPCR) as described in the previous study, no amplicon was detected in 200 ng DNA of the exosome-treated monocyte. The limit of detection was 0.2 ng of Namalawa cells, which harbor 1 copy per genome (two copies per cell) [17].

### 3.5. Immunohistochemical Detection of Dendritic Cells in Gastric Cancer Tissue

The clinicopathological parameters of gastric cancer cases used in this study are summarized in Table 3.

**Figure 4:**
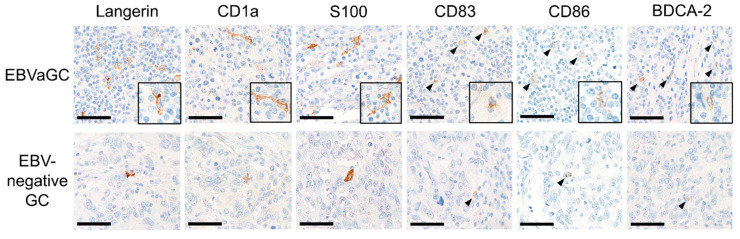
Representative figures of dendritic cells expressing each DC marker in gastric cancer tissue. DCs expressing Langerin, CD1a, and S100 were identified as cells with abundant dendrites. DCs expressing BDCA-2, CD83, and CD86 were identified as relatively small round cells (arrowhead). More abundant infiltration of dendritic cells in EBVaGC was observed than in EBV-negative gastric cancer for each DC marker. Immunohistochemical analysis of Langerin, CD1a, S100, CD83, CD86 and BDCA. Scale bar: 50 µm.

Dendritic cells expressing Langerin, CD1a, S100, CD83, CD86, and BDCA-2 were detected in gastric cancer tissue by immunohistochemical analysis (Figure 4). Dendritic cells expressing Langerin, CD1a, and S100 were identified as cells with abundant dendrites. Dendritic cells expressing BDCA-2, CD83, and CD86 were recognized as relatively small round cells. In EBVaGC, the dendrites of Langerin, CD1a, and S100 positive cells were more prominent compared to EBV-negative GC. Dendritic cells expressing Langerin, CD1a, S100, and BDCA-2 were homogeneously distributed in the tumors, although dendritic cells expressing CD83 and CD86 tended to aggregate in areas with abundant lymphocytes.

**Figure 5:**
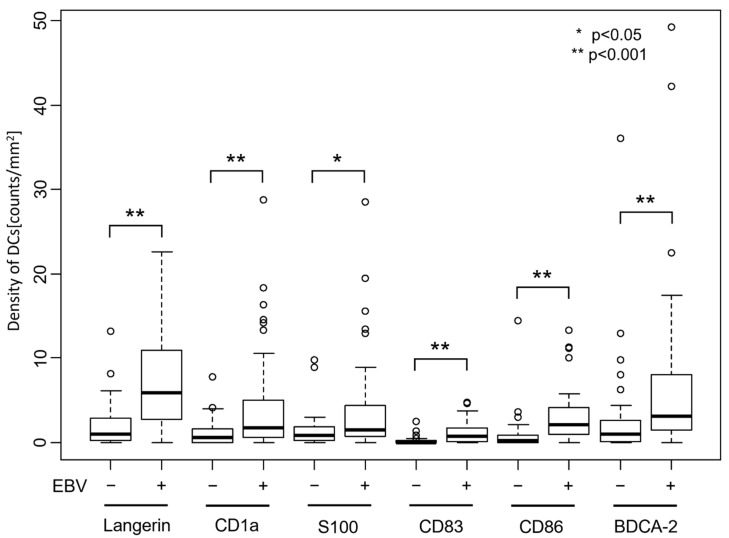
Number of dendritic cells that infiltrated gastric cancer tissue. Dendritic cells expressing each DC marker were counted, and the number per tumor area is shown. The number of DCs is significantly higher in EBVaGC than in EBV-negative gastric cancer for any of the DC markers (*p* < 0.001 for Langerin, CD1a, CD83, CD86, and BDCA-2. *p* < 0.05 for S100, determined by using Wilcoxon rank sum test).

The number of dendritic cells per tumor area was significantly higher in EBVaGC cases than in EBV-negative GC cases for any of the DC markers (Figure 5, *p* < 0.001 for Langerin, CD1a, CD83, CD86, and BDCA-2, and *p* < 0.05 for S100, respectively, determined by using a Wilcoxon rank sum test).

### 3.6. Correlation between Density of Dendritic Cells and Clinicopathological Factors

The correlation between the density (number of dendritic cells per tumor area) and clinicopathological factors were evaluated among EBV-negative GC and EBVaGC; marked differences in both types of gastric carcinoma were observed (Table 4). In EBV-negative cases, the high density of DCs expressing CD1a was correlated with advanced (pT2–4) T stage, venous invasion, lymphatic invasion, and lymph node metastasis (*p* < 0.05). On the other hand, in EBVaGC, the high density of DCs expressing CD1a was correlated with the absence of lymphatic invasion (*p* < 0.05). The high density of DCs expressing CD83 was correlated with early (pT1) T stage (*p* < 0.05). Furthermore, the high density of DCs expressing BDCA-2 was correlated with diffuse histology and advanced (pT2–4) T stage (*p* < 0.05).

**Figure 6:**
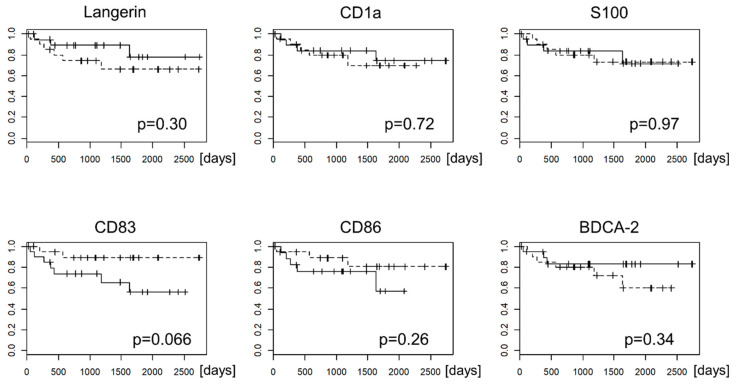
Kaplan–Meier curve of overall survival of patients with EBVaGC. Gastric cancer cases were divided into two groups according to the number of infiltrating DCs that express the indicated DC markers. The cases with less CD83-positive DCs tended to have worse prognosis, although this was not statistically significant (*p* = 0.06, log-rank test).

For the comparison of the prognosis of EBV-negative GC and EBVaGC, gastric cancer cases were divided into DC-high and DC-low cases, and overall survival was compared using the Kaplan–Meier method (Figure 6). In EBVaGC, overall survival tended to be worse in cases with low CD83-positive DCs and CD86-positive DCs than in cases with high CD83-positive DCs and CD86-positive DCs, although the difference was not significant (*p* = 0.066 and 0.26, respectively, determined by using log-rank test). In EBV-negative GC, no significant difference in overall survival was observed between DC-high and DC-low cases (data not shown).

### 3.7. Assessment of Mature DC Ratio in DC-High and DC-Low EBVaGC Cases

**Figure 7:**
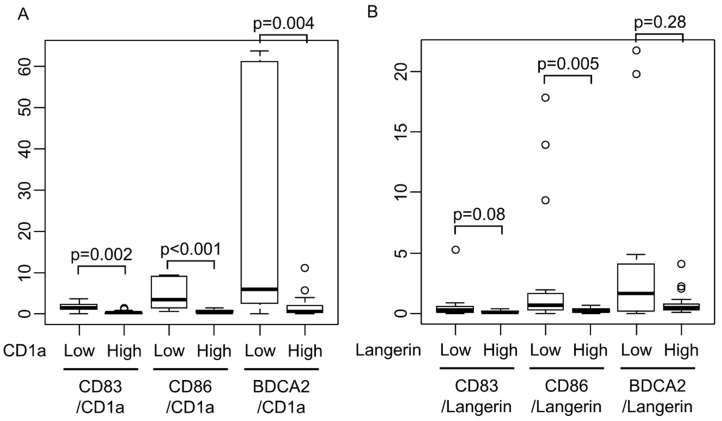
Mature DC ratio in DC-high and DC-low cases. CD83/CD1a ratio and CD86/CD1a ratios were significantly lower in CD1a-high cases than in CD1a-low cases (**A**, *p* < 0.01, determined by using Wilcoxon rank sum test). The similar result was also obtained in Langerin (**B**, *p* = 0.08 and *p* = 0.005, respectively, Wilcoxon rank sum test).

The transition to mature dendritic cells in EBVaGC was assessed in detail, and CD83/CD1a ratio and CD86/CD1a ratios were determined to be significantly low in the group with abundant CD1a-positive dendritic cells (Figure 7A, *p* < 0.01, determined by using Wilcoxon rank sum test). A similar result was also obtained in Langerin (Figure 7B, *p* = 0.08 and *p* = 0.005, respectively, determined by using Wilcoxon rank sum test).

## 4. Discussion

Much attention has been focused on exosomes as tools in intercellular communication that contribute to the establishment of the tumor microenvironment. It is becoming evident that EBV infection alters exosome contents, in addition to its capacity to incorporate virus-derived gene products [18]. However, little is known about the secretion and composition of exosomes from EBVaGC. As a first step in investigating EBVaGC exosomes, we analyzed exosomes from gastric cancer cell lines with and without artificial EBV-infection. We found a marked change in exosome marker proteins (CD63 and CD81) in both cellular production and exosome composition. An increase in the amount of CD63 and CD81 with a decrease in protein amount and particle size suggests that EBV infection considerably affects the dynamics of exosome secretion. Furthermore, in comparison with the amount of CD63 and CD81 proteins in EBV-negative gastric cancer cell lines, an increase in the amount of CD63 and CD81 protein in SNU719, which is an EBVaGC cell line, was observed, indicating an increase in exosome delivery to the microenvironment. Lio et al. [19] recently demonstrated that LMP1, a latent gene protein of EBV, promotes the secretion of exosomes in EBV-positive nasopharyngeal carcinoma. EBV may also control exosome secretion mechanisms in the infected cells of gastric carcinoma. It is worth noting that during lytic infection and egress of EBV, one of lytic gene products, BFRF1, recruits the endosomal sorting complex required for transport (ESCRT) to the nuclear envelope, which is involved in biogenesis of exosomes [20]. However, lytic infection process was not observed in EBV-infected gastric cancer cell lines and SNU719.

DCs are central to tumor immunity [4]. To prove that EBV-infected epithelial cells change the function of DCs through exosomes, flow cytometry analysis of moDCs was performed after culturing blood monocytes with gastric cancer-derived exosomes. CD86-positive moDC fraction was decreased in cultures with exosomes derived from MKN74 + EBV and SNU719 compared to that with exosomes derived from MKN74, indicating that EBV-infection imposes a negative effect on the induction of tumor immunity via their exosomes. Pegtel et al. [5] demonstrated that EBV-miRNAs in the exosomes from EBV-infected B cells are internalized by non-infected moDCs, resulting in the downregulation of immunoregulatory genes. Further studies are necessary to identify the constituents of EBVaGC exosomes, including EBV-miRNAs [18].

In the present study, we used dendritic cell markers suitable for immunohistochemical analysis to visualize tumor-associated dendritic cells (TA-DCs) in the tissue specimens. Langerin and CD1a were used as markers for immature moDCs and CD83 and CD86 for mature or activated DCs. S100 has been used as a general marker for DCs, and BDCA-2 is relatively specific to plasmacytoid DCs (pDCs) [11]. Here we show that the number of DCs was higher in EBVaGC than in EBV-negative GC with any of the markers. Our results are consistent with those in previous studies on EBVaGC [7,8] and nasopharyngeal carcinoma [21,22]. Furthermore, we demonstrated that the significance of DCs is different between GC with and without EBV infection. As shown in Table 4, a significantly higher number of CD1a-positive DCs was observed in the clinical parameters of tumor invasion in EBV-negative GC, but such a correlation was not identified in EBVaGC. The number of CD1a-positive DCs was inversely higher in cases without lymphatic invasion. This shows the potential alteration of biological behavior in CD1a-positive DC, although further investigations are needed.

CD83-positive DCs were significantly lower in gastric carcinomas, showing deep infiltration. This result is in accordance with a report of van Beek et al. wherein CD83-positive dendritic cells were reportedly increased in EBVaGC in comparison to EBV-negative GC and among cases without lymph node metastasis [9]. As for the prognosis analysis, the cases with low numbers of CD83- and CD86-positive DCs showed a relatively worse prognosis than those with a high number of the same DCs. This result is concordant with that of Gong et al., who used fascin as a marker of mature dendritic cells [10], indicating that suppression of dendritic cells contributes to the progression of EBVaGC. Importantly, the proportion of CD83- and CD86-positive DCs to Langerin- or CD1a-positive DCs were significantly low in the group with a high number of Langerin- or CD1a-positive DCs. These findings indicate that there are some mechanisms that suppress the maturation of immature DCs in EBVaGC and correspond to the results of in vitro experiments on exosomes, i.e., a decrease in CD86-positive population by exosomes derived from EBV-positive gastric cancer cell lines.

The significance of BDCA-2-positive DCs is noteworthy. BDCA-2 is relatively specific to pDCs, whose origin is different from that of conventional DCs [23]. In the present study, a high number of BDCA-2-positive DCs was correlated with tumor invasion depth and was inversely proportional to the number of CD1a-positive cells in EBVaGC. However, the ratio of BDCA-2-positive DCs to immature DCs was similarly low in the group with a high number of Langerhans- and CD1a-positive DCs. Further studies are necessary to clearly elucidate the interrelationship among DC subtypes in EBVaGC.

Several limitations in the present study should be noted. We did not evaluate the profiles of exosome cargoes (especially viral proteins, non-coding RNAs, and microRNAs) and their relation to cellular counterparts. Specific subsets of EBV-lytic genes were consistently expressed in EBVaGC, although their function and their incorporation to the exosomes have not been clarified [24]. Furthermore, we focused on the tumor microenvironment, but EBV-exosome research encompasses the whole process of carcinogenesis, from the infection of epithelial cells to full-blown carcinoma. In this regard, Nanbo et al. demonstrated the contribution of exosomes in cell-to-cell viral transmission [25].

## 5. Conclusions

EBVaGC is a tumor with abundant DCs, including immature and mature moDCs and pDCs. Moreover, the maturation of DCs is suppressed, at least in part, by exosomes from EBV-infected epithelial cells.

## Figures and Tables

**Table 1:** Effect of artificial EBV-infection.

	Protein (µg/105 Cell) Fold	CD63 Density Fold	CD81 Density Fold	Size
Cell lysate				
MKN7 + EBV/MKN7		2.147	1.051	
MKN74 + EBV/MKN74		4.046	2.973	
Exosome				
MKN7 + EBV/MKN7	0.505/0.515			not applicable
	0.981	2.104	1.601	
MKN74 + EBV/MKN74	0.310/0.380			33.9 nm/71.3 nm
	0.8158	2.829	2.878	0.475

Total protein concentration of exosome sample was measured by BCA assay and adjusted against the original cell number. CD63 and CD81 density was obtained by the densitometry of Western blots. The size of exosomes was obtained by electron microscopy. Not applicable: electron microscopy was not performed for MKN7 or MKN7 + EBV.

**Table 2:** Comparison of protein concentration and exosome size between EBVaGC and EBV-negative GC cell lines.

	PROTEIN (µG/10^5^ Cell) Fold	Cd63 Density Fold	CD81 Density Fold	Size
Cell lysate				
SNU719/MKN7		2.617	1.032	
SNU719/MKN74		2.005	3.324	
Exosome				
SNU719/MKN7	2.29/0.515			not applicable
	4.447	4.844	2.504	
SNU719/MKN74	2.29/0.380			69.6 nm/71.3 nm
	6.026	8.785	5.605	0.976

The meaning of each value is the same as Table 1. not applicable: electron microscopy was not performed for MK7.

**Table 3:** Clinicopathological characteristics of gastric cancer cases used for this study.

Variables	EBV-Negative GC	EBVaGC
	N = 40	N = 41
Age (y)		
Median (range)	65 (33–82)	69 (41–87)
Sex		
Female	7 (17.5)	6 (14.6%)
Male	33 (82.5%)	35 (85.4%)
Size (cm)		
Median (range)	5.1 (0.3–16.0)	4.5 (1.3–12.5)
Location		
Upper	5 (12.5%)	22 (53.7%)
Middle	21 (52.5%)	17 (41.5%)
Lower	14 (35.0%)	2 (4.9%)
Lauren classification		
Diffuse	18 (45.0%)	20 (48.8%)
Intestinal	22 (55.0%)	21 (51.2%)
Tumor depth		
Early	21 (52.5%)	24 (58.5%)
Advanced	19 (47.5%)	17 (41.5%)

**Table 4:** Correlation between the number of dendritic cells per tumor area and clinicopathological factors.

		EBV-Negative GC						EBVaGC					
		Langerin	CD1a	S100	CD83	CD86	BDCA-2	Langerin	CD1a	S100	CD83	CD86	BDCA-2
Age	<65	2.71	1.30	1.21	0.13	1.32	2.88	9.98	5.40	5.96	1.58	3.01	6.49
	≥65	1.16	1.09	1.69	0.27	0.49	2.64	7.10	4.56	3.29	0.90	3.29	7.71
Sex	Male	1.75	1.29	1.64	0.23	1.01	2.84	8.42	4.84	4.18	1.16	2.51 *	6.25
	Female	2.61	0.69	0.61	0.07	0.50	2.37	6.58	5.07	4.79	1.10	6.65 *	13.19
Size	<45 mm	2.92	0.71	1.80	0.24	0.57	3.94	10.60	6.17	5.34	1.36	3.58	7.82
	≥45 mm	1.22	1.50	1.24	0.18	1.16	1.96	5.81	3.63	3.24	0.95	2.84	6.74
Lauren classification	Intestinal	2.22	0.75	1.81	0.19	0.63	2.52	7.00	5.42	3.63	1.05	3.61	4.73 *
	Diffuse	1.51	1.72	1.04	0.21	1.25	3.04	9.36	4.30	4.93	1.25	2.78	9.93 *
Tumor depth	Early	1.92	0.50 *	1.92	0.18	0.49	3.05	9.35	5.12	4.31	1.56 *	3.37	5.45 *
	Advanced	1.88	1.94 *	0.96	0.22	1.37	2.43	6.46	4.51	4.20	0.58 *	2.90	9.82 *
Venous invasion	Absent	2.08	0.54 *	1.39	0.30	1.15	3.81	7.87	4.91	4.04	1.27	4.14	8.39
	Present	1.68	1.97 *	1.56	0.09	0.64	1.46	8.35	4.84	4.43	1.07	2.53	6.47
Lymphatic invasion	Absent	2.02	0.45 *	1.83	0.18	0.51	1.52	9.58	5.84 *	5.03	1.45	4.03	7.70
	Present	1.78	1.93 *	1.09	0.22	1.34	3.99	5.40	2.99 *	2.79	0.57	1.79	6.42
Lymph node metastasis	Absent	1.40 *	0.44 *	2.03	0.21	0.50	2.98	9.04	5.44	4.78	1.35	3.72	7.12
	Present	2.35 *	1.86 *	0.95	0.19	1.26	2.55	5.74	3.32	2.86	0.61	1.90	7.66

Each value is the average number of dendritic cells per mm^2^ expressing the indicated DC marker. * *p* < 0.05, determined by using Wilcoxon rank sum test. Significant correlations (*p* < 0.05) were identified.

## Appendix A. Estimating the Number of Exosomes

We assumed the following: (1) each exosome is spherical, (2) the total protein content of an exosome is proportional to the volume of the exosome, and (3) the protein content of CD63 and CD81 in an exosome is proportional to the surface area of the exosome. According to the results shown in Table 1, the diameter of the exosome of MKN74 + EBV is approximately 1/2 of the diameter of the exosome of MKN74, 1/4 the surface area 1/, and 1/8 the volume. The total amount of protein in the exosome sample corresponding to 10^5^ cells was approximately 0.8 times, and that of CD63 and CD81 was approximately 2.8 times. Therefore, we estimated that the number of exosomes is approximately 6.4 (=0.8 × 8) to 11.2 (=2.8 × 4) times. In the results shown in Table 2, the diameters of the exosomes in SNU719 and MKN74 were almost equal, but the amount of total protein amount and that of CD63 and CD81 were different by 6–8-fold. Therefore, we estimated the number of exosomes to be different by 6–8-fold.
